# Translation and Validation of the Portuguese Version of the Rating-of-Fatigue Scale

**DOI:** 10.1186/s40798-025-00822-z

**Published:** 2025-02-25

**Authors:** João Barreira, João Brito, Fábio Yuzo Nakamura, Pedro Figueiredo

**Affiliations:** 1 Research Center in Sports Sciences, Health Sciences and Human Development, CIDESD, University of Maia, Maia, Portugal; 2 Portugal Football School, Portuguese Football Federation, Oeiras, Portugal; 3 CIPER, Faculdade de Motricidade Humana, Universidade de Lisboa, Lisboa, Portugal; 4 Physical Education Department, College of Education, United Arab Emirates University, Al Ain, Abu Dhabi, United Arab Emirates

**Keywords:** Recovery, Fatigue Perception, Exertion

## Abstract

**Background:**

The Rating-of-Fatigue (ROF) scale is a validated tool to measure changes in perceived fatigue in sports and everyday contexts; thus, this study aimed to translate and validate the Portuguese version of the ROF scale. For this, the study was composed of three phases. Phase 1 involved a comprehensive translation of the ROF scale into Portuguese, followed by a back-translation and a consolidation process to obtain the final version of the ROF scale in Portuguese. In phase 2, the face validity of the scale was assessed. Seventy-three native Portuguese speakers responded to a series of Likert scale items designed to evaluate the purpose of the scale and assess whether it accurately measured the intended construct. In the final phase, the convergent and divergent validity of the scale was assessed during an incremental cycling test to exhaustion, followed by 10 min of passive recovery and a subsequent repetition of the initial 5 min of the test. The study was conducted between October 2023 and June 2024 in Portugal.

**Results:**

The results from phase 1 indicated a high level of comparability and interpretability between the original and back-translated versions, with only minor adjustments made to finalize the Portuguese version of the ROF scale. In phase 2, a high level of face validity was observed. The median score for the item “measures fatigue” was (median [IQR]) was 3.0 [3.0, 4.0] out of 4. After participants were provided with the scale instructions, the face validity score improved significantly (4.0 [3.0, 4.0]; *p* = 0.001). In phase 3, during the incremental test, very large correlations were observed between ROF, and key physiological and performance indicators, including rating of perceived exertion (RPE), heart rate, oxygen consumption, and power output. Discriminant validity between ROF and RPE was found during the recovery.

**Conclusions:**

The present study indicates that the Portuguese version of the ROF maintains the properties of the original version and can be used effectively in the Portuguese language.

**Supplementary Information:**

The online version contains supplementary material available at 10.1186/s40798-025-00822-z.

## Key Points

The English version of the Rating of Fatigue (ROF) scale demonstrated high levels of face and construct validity in different scenarios and is a valid tool to assess perceived fatigue in multiple daily life contexts.

The ROF scale has not been translated and validated in the Portuguese language yet.

The current study translated and validated the ROF scale to the Portuguese language, maintaining the scale’s original properties, with high levels of face and construct validity.

**Supplementary Information:**

The online version contains supplementary material available at 10.1186/s40798-025-00822-z.

## Background

Fatigue has been described as a complex and multifactorial phenomenon [1, 2]. Over the years, various fatigue dichotomies, such as central and peripheral fatigue, have been developed to better understand and manage this condition [3, 4]. However, they still fail to define the meaning of the concept [5]. Furthermore, these dichotomies are also not capable of distinguishing fatigue from related terms such as somnolence [2], exertion [6], and exhaustion [7], among others.

To date, fatigue is often understood as encompassing two primary dimensions: subjective and objective [5]. Perceived fatigability could be defined as changes in the sensations that regulate the integrity of the performer [8]. Performance fatigability is the decline in an objective measure of performance over a discrete period of time [8]. These constructs are not independent, rather, they interact dynamically to shape the overall fatigue response [8].

Recognizing this complexity, recent efforts have aimed to provide a universal definition of fatigue applicable to both athletic and clinical populations, moving beyond traditional descriptors such as central, mental, muscle, etc. In this context, Enoka & Duchateau [8] defined fatigue as a debilitating symptom of tiredness and weakness, influenced by an interaction between performance fatigability (an acute exercise-induced reduction in force and power output of the involved muscles) and perceived fatigability (changes in sensations accompanying fatigue).

A wide range of tools and methods have been developed to quantify fatigue. This includes self-reported measures, autonomic nervous system function assessments, physical performance tests, and biochemical markers [9–11]. However, logistical constraints, feasibility issues, and concerns about the reliability of some of these methods may impede their regular use [12]. Self-reported measures are widely used to measure fatigue across various populations. Tools such as the Profile of Mood States [13], Recovery Stress Questionnaire [14], Daily Analysis of Life Demands [15], and the Total Quality of Recovery [16] are just a few examples of assessment tools used in the literature. However, most of these tools share some practical limitations, such as length, narrow focus, or lack of specificity. This has led practitioners and researchers to develop and incorporate customized shortened questionnaires into their monitoring practices and research. However, the reliability and sensitivity of these customized tools were not established [17].

Micklewright et al. [18] recently developed and validated a general “Rating-of-Fatigue” (ROF) scale. This 11-point scale, accompanied by descriptive anchors, is intended to assess fatigue across diverse settings, such as daily life, physical activity, and recovery. The ROF scale has shown strong face and construct validity in evaluating fatigue during ramped exercise, resting recovery, and variations influenced by circadian and circaseptan (weekly) rhythms. Additionally, the scale demonstrated discriminant validity from the Rating of Perceived Exertion (RPE) scale [6] during post-exercise recovery, and it correlated well with physiological markers, such as oxygen consumption and heart rate, during the recovery period. Previous research [19, 20] has used RPE to monitor post-exercise recovery, highlighting the differences between exertion and fatigue and emphasizing that RPE should not be used to assess momentary fatigue. Overall, the ROF scale offers practical advantages over other self-reported fatigue measurements, including its quick response time, ease of use, and ability to capture momentary fatigue without relying on participants to recall their fatigue levels over predefined periods. Thus, the ROF scale is a promising instrument for measuring fatigue, and some studies have already utilized it [21–23].

Given the wide range of potential applications of the ROF scale, it is important to translate the instrument and validate it in other languages. As highlighted by Brownstein et al. [24], simply translating a scale from one language to another without accounting for potential cross-cultural and ethnic differences is considered inadequate. Therefore, it is recommended that, when translating scales and questionnaires, a thorough process of translation, back-translation, and consolidation be conducted [25]. Additionally, testing for face and construct validity is recommended to ensure that the translated version retains the validity and measurement properties of the original version, as required for the intended application [25]. Portuguese is ranked 6th among the most spoken worldwide, with approximately 236 million native speakers [26] distributed across multiple countries, including Portugal, Brazil, Angola, and Mozambique. Although these countries share the Portuguese language, some linguistic and cultural variations exist. The purpose of the present study was to translate and validate the ROF scale to Portuguese, facilitating its use in future research conducted in the Portuguese language. Furthermore, this version of the ROF scale can serve as a starting point for adaptation to various contexts and Portuguese variants, but further cultural tailoring might be required. Such adaptations are crucial to ensure the scale’s validity and reliability across diverse Portuguese-speaking populations.

## Methods

### Design

This study was approved by the Ethics Committee of the Portugal Football School (23/2023) and conducted in accordance with the ethical standards outlined in the Declaration of Helsinki. All participants were Portuguese-native speakers from Portugal and provided informed written consent to participate. The study was divided into three phases to translate and validate the ROF scale in Portuguese: (1) translation, back-translation, and consolidation, (2) face validity testing, and (3) construct and convergent validity testing.

### Phase 1– translation, back-translation, and consolidation

The translation process comprised three sequential stages, following widely recommended guidelines for the cross-cultural adaptation of research instruments [25, 27]. These stages included an initial translation to Portuguese, a back-translation of the Portuguese version into English, and a formal comparison of the back-translated versions with the original ROF scale. For the initial phase, two bilingual native Portuguese speakers, fluent in English who work as professional translators, were asked to independently translate both the ROF scale and its instruction sheet from English to Portuguese. Their translations were reviewed and reconciled by two additional bilingual individuals fluent in English, one of whom was a professional translator. Any inconsistencies were addressed collaboratively to produce a single unified Portuguese version of the scale. Next, the Portuguese version was back-translated into English by two bilingual translators who are native English speakers and proficient in Portuguese. All translators involved were independent of the study authors, unfamiliar with the ROF scale, and unaware of the study’s objectives.

**Table 1:** Comparability/Interpretability rating sheet example. From Sperber et al. [28]

		Extremely Comparable	Moderately Comparable				Not at all comparable		
Original ROF scale [18]	Back-translated English ROF scale version	a) Comparability of language							
		1	2	3	4	5		6	7
		b) Similarity of interpretation							
		1	2	3	4	5		6	7

Based on the method for validating translated instruments developed by Sperber et al. [28] and used by Brownstein et al. [24] to translate the ROF scale and accompanying instructions into French, the original ROF scale and instructions in English was compared with the two back-translated versions to examine any discrepancies. This procedure involved using the “Comparability/Interpretability Rating Sheet” [28] (Table 1). Each item (phrase) in the two back-translated English versions of the scale was ranked for comparability of language and similarity of interpretability against the original ROF scale. Comparability of language refers to the formal similarity of the words, phrases, and sentences. In contrast, similarity of interpretability assesses the extent to which the two versions convey the same response, even if the wording differs. A Likert scale ranging from 1 (extremely comparable/extremely similar) to 7 (not at all comparable/not at all similar) was used to assess both comparability and interpretability. A panel of four native English speakers conducted the formal translation review using the Comparability/Interpretability Rating Sheet. If an average score for comparability and interpretability exceeded 3, corrections to the translated ROF version were made [28]. After these revisions, the Portuguese version of the ROF scale, as well as the accompanying instructions, was finalized (Supplementary File 1).

### Phase 2– face validity testing with the portuguese rating-of-fatigue scale

To assess face validity, methods adapted from Micklewright et al. [18] and Brownstein et al. [24] were employed, adhering to established guidelines [29]. Seventy-three native Portuguese-speaking participants from Portugal were recruited for this study phase (age: 22.2 ± 3.0 years old; height: 176.9 ± 8.8 cm; mass: 72.7 ± 12.8 kg). Participants included Sports Sciences Bachelor students (*n* = 35), Sports Sciences master students (*n* = 30), Sports Sciences doctoral students (*n* = 7), and Sports Sciences academics (*n* = 1), providing a range of expertise levels in the field of sport and exercise. Face validity, a subjective measure of whether the tool evaluates its intended construct, was tested by asking participants to rate what they believed the ROF scale measured. Participants were asked to rate what they believed the ROF scale measured by responding to five questionnaire items. Responses were rated on a 5-point Likert scale ranging from 0 (Strongly Disagree) to 4 (Strongly Agree). The questions aimed to assess the extent to which the ROF scale: (i) represents fatigue, (ii) represents exertion, (iii) has descriptive components that make the scale easy to understand (iv) has descriptive components that assist in deciding on a rating, and (v) is overall difficult to understand. These questions were derived from the original ROF scale validation by Micklewright et al. [18]. The three questions regarding the diagrammatic components and visual appearance were omitted, as they were deemed irrelevant to the translation and subsequent interpretation. This approach aligns with the face validity testing phase of the French ROF Scale translation and validation process [24]. Participants then read the instruction sheet that accompany the scale and repeated the ratings, including an additional question on the instructions’ usefulness.

### Phase 3– convergent and discriminant validity testing with the portuguese rating-of-fatigue scale during a step cycling protocol to exhaustion and recovery

In the final phase of the translation and validation process of the Portuguese version of the ROF scale, construct validity was assessed. For this phase, 21 college students from Portugal (10 female; age: 26.4 ± 4.7 years old; height: 169.8 ± 9.7 cm; weight: 65.7 ± 13.1 kg, ) were recruited. Inclusion criteria for this phase were: age between 18 and 40 years old, and being free from any neurological, rheumatological, cardiovascular, respiratory, or metabolic diseases, traumatic injuries, or functional impairment affecting cycling.

Participants completed a step cycling protocol to exhaustion on a cycle ergometer (Excalibur Sport, Lode, The Netherlands) and then remained seated on the cycle ergometer for 10 min while recovery measurements were taken. Before the test, participants self-reported their physical activity levels. The starting power output was 60 W for women and 80 W for men, with a step increment of 20 W every 2 min for women and 30 W every 2 min for men. Participants were instructed to maintain a pedal rate between 70 and 85 rotations per minute (RPM). Failure to stay within this RPM range or voluntarily stop pedaling despite strong verbal encouragement were considered exhaustion. Throughout the protocol, heart rate (HR; Polar H10 chest strap, PolarElectro Oy, Kempele, Finland), oxygen consumption (VO_2_; MetaLyzer 3B, Cortex) [30], power output, and ROF and RPE were recorded every 100 s. During recovery, HR, VO_2_, ROF, and RPE were recorded every 120 s up to 10 min. The RPE score was derived from the Borg 6–20 scale [6]. Two objective testing methods were used during both ramped exhaustive cycling exercise and 10 min of recovery: (i) convergent validity, which involved calculating correlations between ROF and HR, power output, and RPE; (ii) discriminant validity, which measured the degree to which ROF and RPE diverged. After 10 min of recovery, participants repeated the first 5 min of the step cycling protocol, with the same measurements taken at the 01:44, 03:20, and 5:00 min time points, as performed in the initial protocol.

### Statistical analysis

Statistical analyses were performed using R statistical software (version 4.2.2, R Foundation for Statistical Computing, Vienna, Austria). For face validity, all Likert scale questionnaire responses were recorded from 0 to 4, with 0 representing low face validity and 4 representing high face validity for question 1 (scale measures fatigue). The scores in response to the five questions given before and after the administration of the instruction sheet were compared using non-parametric Wilcoxon’s signed rank test. To quantify the magnitude of these changes, we calculated the Probability of Superiority (A_w_) using the formula:

$$\:A\omega\:=\:\frac{Number\:of\:positive\:differences\:+\:0.5\:x\:Number\:of\:ties}{Total\:number\:of\:pairs}$$

The A_w_ measure provides an estimate of the probability that a randomly chosen post-treatment observation is greater than a randomly chosen pre-treatment observation [31]. The conventional thresholds for small, moderate, and large effect sizes in d (i.e., 0.20, 0.50, and 0.80) can be converted to the Aw-metric (i.e., 0.56, 0.64, and 0.71) [31]. For the convergent validity testing, all variables were expressed relative to the percentage of time to exhaustion, with 0% representing the beginning of the step cycling protocol and 100% representing the point of volitional exhaustion. To provide a continuous scale during recovery, recovery time was also expressed as a percentage of time to exhaustion, whereby the point of fatigue is at 100%, and recovery time is expressed as a percentage increase relative to the time to exhaustion [18]. Measures of oxygen consumption and HR were averaged for the last 15 s of every 100 s interval during exercise and post-recovery and for the last 15 s of every 120 s interval during recovery. To assess the degree of association between ROF and RPE, HR, VO_2_, and power output during exercise and the recovery period, within-subject correlations were used for each physiological measurement against ROF using the *rmcorr* package. Correlation coefficients were qualitatively interpreted as follows: trivial (*r* ≤ 0.1), *small* (*r* = 0.1–0.3), *moderate* (*r* = 0.3–0.5), l*arge* (*r* = 0.5–0.7), *very large* (*r* = 0.7–0.9) and *almost perfect* (*r* ≥ 0.9) [32].

To compare reported ROF and RPE, HR, and VO_2_ during the first 5 min of the step cycling protocol with the post-recovery sessions, a linear mixed model analysis was employed. ROF, RPE, HR, and measures of oxygen consumption were treated as dependent variables. Test (initial or post-recovery) and time-point (01:44, 03:20, and 5:00 min) were considered fixed effects (with an interaction), and subject ID was included as a random effect. When significance was observed for the main effects, post-hoc pairwise comparison tests using Bonferroni correction were conducted to assess differences between test timings for the same time-point.

## Results

### Phase 1– translation, back-translation, and consolidation

The Portuguese version of the ROF scale was obtained through two independent translators, which were then reviewed and consolidated by two other native Portuguese speakers, fluent in English, one being a professional translator. This consolidated version was back translated into English by two additional translators, and comparisons were made between the back-translated versions and the original ROF scale. Four sentences of one of the back-translated versions received an average comparability rating > 3, and three sentences from the same back-translated version received an average interpretability > 3. The main issues were the use of the word “tired” instead of “fatigued” on the back-translated version, which appeared in both the instructions sheet and the scale itself, and a misspell of the word “two” as “to”. The average scores for comparability and interpretability of the back-translated version 1 were 1.4 and 1.2, respectively, for the instructions sheet, and 1.3 and 1.2, respectively, for the scale itself. The average scores for comparability and interpretability of the back-translated version 2 were 2.3 and 1.9, respectively, for the instructions sheet, and 2.4 and 1.8, respectively, for the scale itself. Altogether, the average comparability and interpretability scores were 1.9 and 1.6 for the instructions sheet and 1.9 and 1.5 for the scale itself. Minor modifications were thus made to the Portuguese version.

### Phase 2– face validity testing with the portuguese rating-of-fatigue scale

**Fig. 1:**
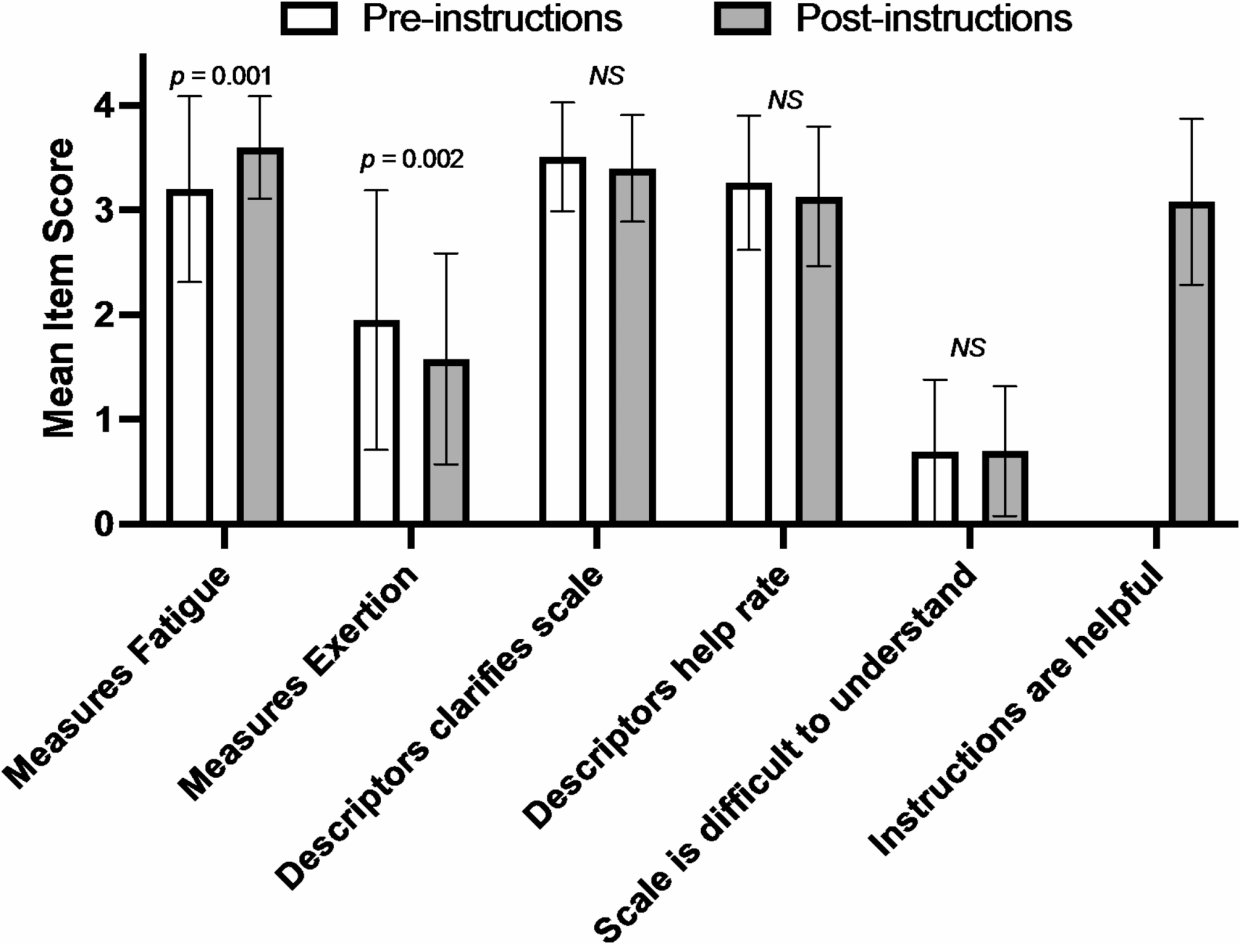
Face validity outcomes of the rating-of-fatigue scale before and after the scale instructions. Abbreviations: NS, non-significant

Scores for the face validity Likert questionnaires are presented in Fig. 1 and results are presented as median (interquartile range [IQR]). A high level of face validity was indicated by the high median Likert scores for question 1 (scale measures fatigue). The score for this question increased further after reading of the instructions (pre: 3.0 [3.0, 4.0] vs. post: 4.0 [3.0, 4.0]; *p* = 0.001; A_w_ = 0.59), indicating that the instructions were effective in clarifying the purpose of the scale. Likert scores indicated that participants were initially undecided about whether the scale represented exertion (2.0 [1.0, 3.0]), but this improved after reading the instructions (1.0 [1.0, 2.0]; *p* = 0.002; A_w_ = 0.40]). Descriptors were perceived as helpful in clarifying the scale, with slight decrements in mean Likert scores from pre- to post-instructions, which were not significant (4.0 [3.0, 4.0] vs. 3.0 [3.0, 4.0]; *p* = 0.09; A_w_ = 0.44). These descriptors were also perceived as helpful when deciding how to rate the scale, with slight, non-significant decrements in mean Likert scores from pre- to post-instructions (3.0 [3.0, 4.0] vs. 3.0 [3.0, 4.0]; *p* = 0.16; A_w_ = 0.46). Participants perceived the scale as easy to understand, as indicated by the low scores for the item “scale is difficult to understand (1.0 [0.0, 1.0] vs. 1.0 [0.0, 1.0]; p = 0.06; A_w_ = 0.52). Lastly, a score of 3.0 (3.0, 4.0) was obtained for the item “instructions are helpful”.

### Phase 3– convergent and discriminant validity testing with the portuguese rating-of-fatigue scale during the step cycling protocol to exhaustion and recovery

**Table 2:** Within-subject correlation coefficients and associated 95% confidence interval (CI) with the rating-of-fatigue scale during step cycling protocol and 10-minutes of passive seated recovery

	Within-subject correlations		
	*r*	95% CI	*p*
*Graded exercise*			
Rating of perceived exertion	0.98	0.97, 0.99	< 0.001
Heart Rate	0.91	0.89, 0.93	< 0.001
Oxygen Consumption	0.89	0.85, 0.93	< 0.001
Power Output	0.93	0.91, 0.94	< 0.001
*10-minute passive recovery*			
Heart Rate	0.73	0.65, 0.79	< 0.001
Oxygen Consumption	0.45	0.31, 0.60	< 0.001

**Fig. 2:**
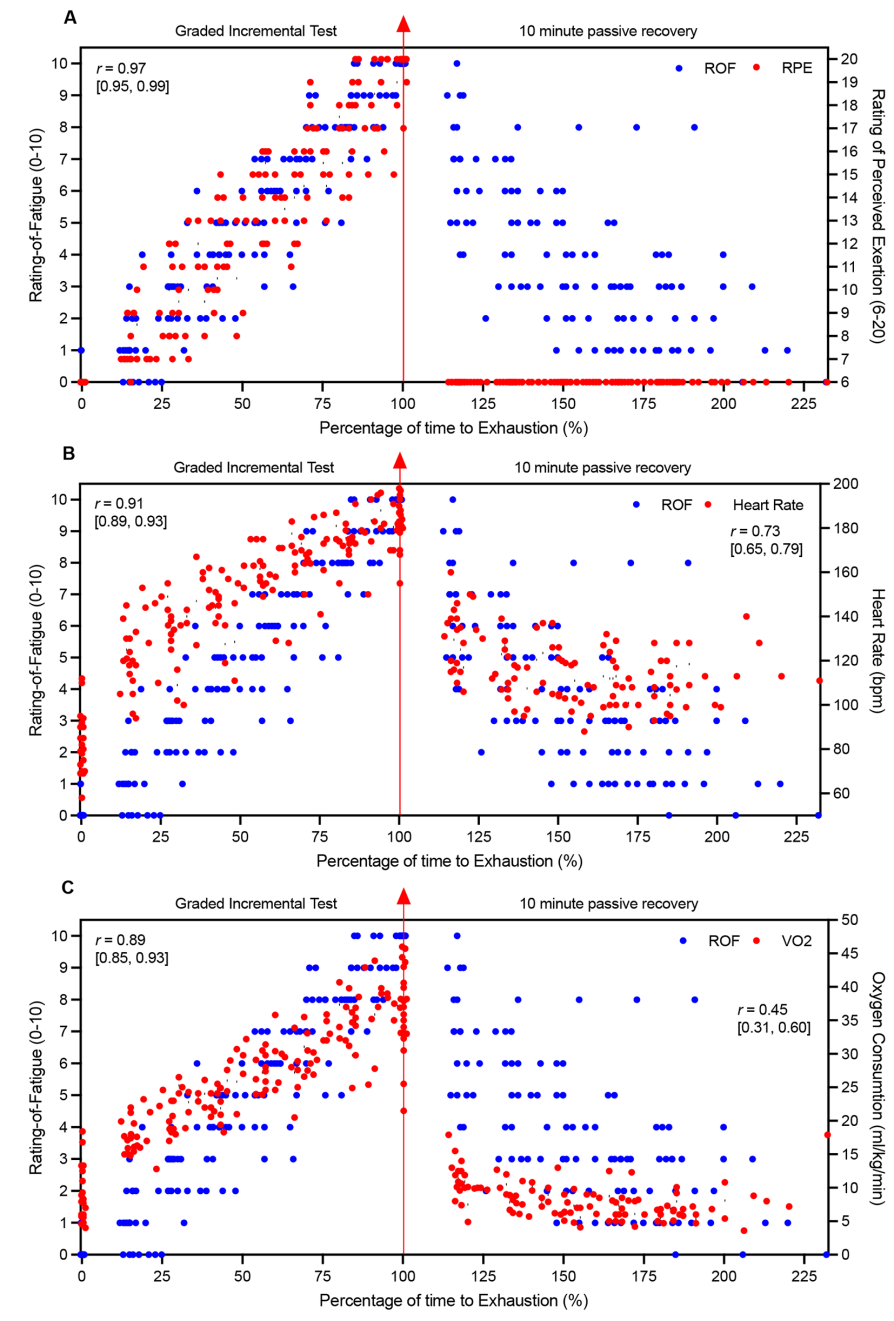
Relationship between ratings of fatigue and perceived exertion (**A**), heart rate (**B**), and oxygen consumption (**C**) during the step cycling protocol and 10 min of seated passive recovery. Each dot represents an individual observation

During the step cycling protocol, very large correlations were observed between ROF, RPE, HR, VO_2_, and power output (Table 2). Similarly, a very large correlations was found between ROF and HR, and a moderate correlation between ROF and VO_2_ during the 10-minute seated passive recovery period (Table 2). However, during recovery, ROF and RPE could not be correlated as RPE scores were uniformly 6, showing no variance. The within-subject correlations between ROF and HR, RPE, VO_2_, and power output during the graded test and recovery are shown in Fig. 2.

**Fig. 3:**
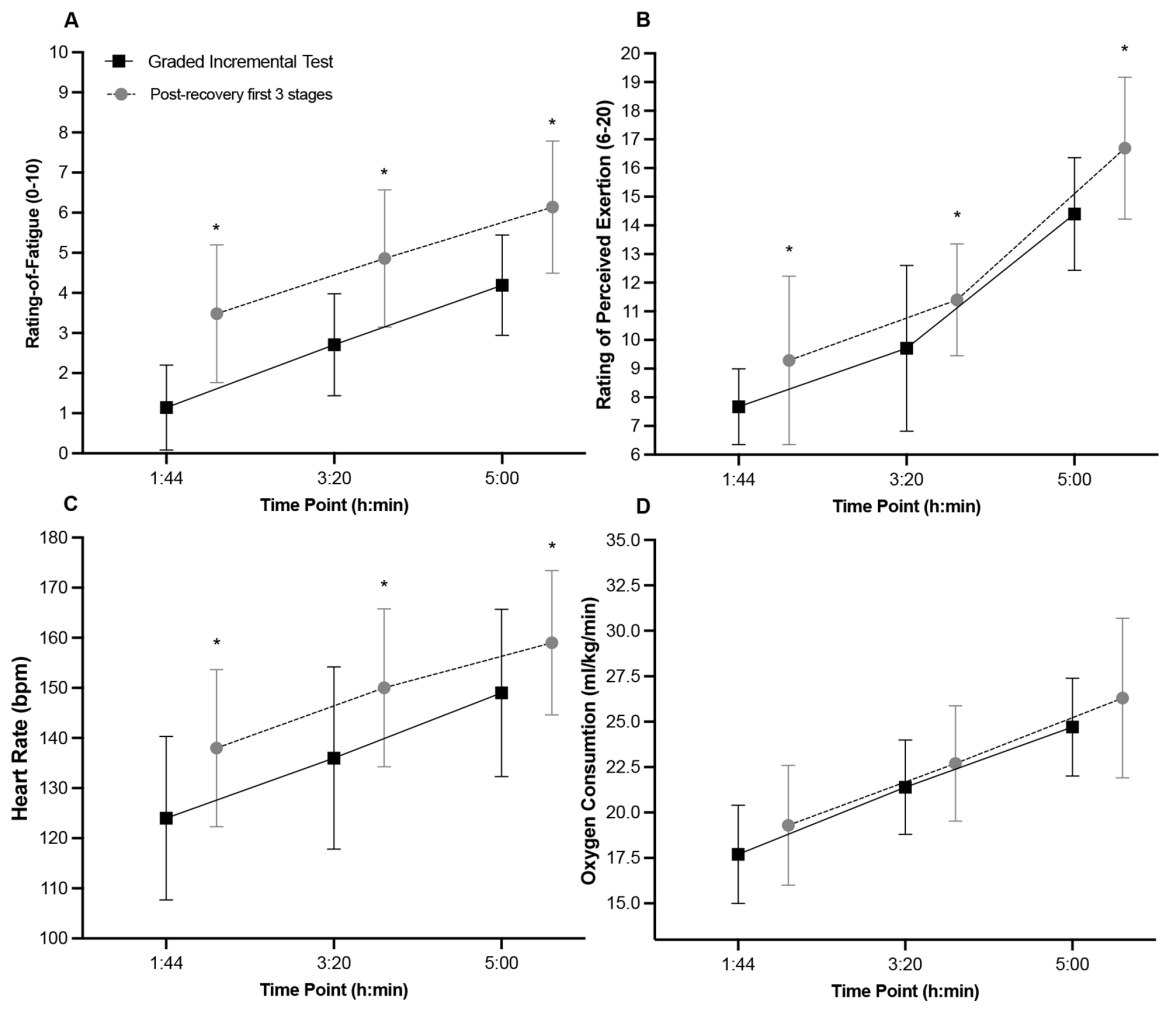
Rating of fatigue (**A**), RPE (**B**), heart rate (**C**), and oxygen consumption (**D**) at the first 3 time-points during the initial step cycling protocol (black) and the repetition of the first 5-minutes of the protocol after the 10-min recovery (grey). * Indicates a significant difference from the step cycling protocol at the same time point

## Discussion

The study aimed to validate the ROF scale in Portuguese by following recommended guidelines for cross-cultural adaptation of study instruments [25, 28], and also by using the steps employed by Brownstein et al. [24] for translating the ROF scale into French. Following the translation and back-translation process, comparability and interpretability were considered acceptable for the ROF scale and the accompanying instructions, with scores similar to those obtained by Brownstein et al. [24].

Some minor modifications to the final scale and instructions were made. Specifically, the word “tired” (*cansado* in Portuguese) appeared instead of “fatigued” on the back-translated version, both in the scale itself and in the instruction sheet. Additionally, there was a misspelling of the word “two” (it was presented as “to” instead of “two”). Thus, these issues were promptly corrected for the final version of the scale and the instruction sheet.

Even though the words “tired” and “fatigued” are distinct in Portuguese, their meaning could be conflated. While the concepts are related, they have different definitions in English. The term “tired” lacks a specific definition in sports sciences, but the Oxford English Dictionary (2011) defines tiredness as “the state of wishing for sleep or rest”. In contrast, fatigue is described as a debilitating symptom of tiredness and weakness, dictated by an interaction between performance fatigability, involving an acute exercise-induced reduction in force and power output of the involved muscles and perceived fatigability, involving changes in sensations that accompany fatigue, as defined earlier [8]. While tiredness is a component of fatigue, it is not synonymous with fatigue. Interestingly, Brownstein et al. [24] encountered a similar issue with the French back-translated versions of the ROF scale. However, while the words for “fatigued” and “tired” are the same in French (*fatigué*), the same does not apply to Portuguese, where “tired” is “*cansado*” and “fatigued” is “*fatigado*”. Thus, this issue may reflect a lack of knowledge among the translators regarding the differences between these constructs, which are often used interchangeably, as mentioned before. However, we acknowledge that the descriptions in the accompanying instructions for the upper anchoring of the scale may lead to confusion between “fatigue” and “tiredness”. Specifically, “…situations such as sprinting until you can no longer physically continue” may merge both constructs. Properly anchoring the upper value of a scale is of great importance, as it may greatly impact the ratings [33]. As such, considering that two examples are provided for the upper anchor, we recommend future users of the scale to use or provide only one of them, depending on the intended use of the scale, to avoid any confusion.

In phase 2 of the study, the face validity of the Portuguese-translated ROF scale was assessed using Likert questionnaires, employing methods similar to those in the original validation study of the ROF scale [18] and the validation of the French version [24]. This approach adhered to established guidelines for validity testing [29]. The scale exhibited a high level of face validity, as evidenced by the high score on the item assessing “whether the scale measures fatigue”. This score was further improved by the accompanying instructions sheet, which clarified the scale’s purpose. Consequently, it is recommended that the instructions be provided alongside the scale to facilitate comprehension and ensure accurate use, with scores decreasing further after the presentation of the instructions sheet. This indicates that participants were able to differentiate between the constructs of fatigue and exertion, as previously demonstrated by Micklewright et al. [18]. While fatigue and exertion are closely associated and highly correlated during the incremental test, they diverge during the recovery period. Specifically, fatigue remains elevated but decreases over the 10-minute recovery period, whereas perceived exertion immediately declines as participants are not engaged in any physical effort. Furthermore, fatigue can also be accumulated throughout the day from various activities, and it may be perceived even at rest [18]. Lastly, the descriptors accompanying the scale were reported as helpful in clarifying the scale’s purpose and assisting participants in selecting an appropriate response.

Finally, in phase 3, the construct validity of the Portuguese ROF scale was assessed during a step cycling protocol. Similarly to the results of Micklewright et al. [18] and Brownstein et al. [24], ROF was found to be correlated with RPE, HR, and VO_2_ measurements during the incremental protocol. During the recovery period, an expected divergence between ROF and RPE was observed, which underscores that the ROF scale maintains discriminant validity from perceived exertion. This finding aligns with the results demonstrated in the other two validation studies [18, 24]. Interestingly, and in line with the other two validation studies, an almost linear decrease in perceived fatigue during recovery was observed. However, the correlations with physiological measures were not as strong as those reported by Micklewright et al. [18] and Brownstein et al. [24].

First, it must be acknowledged that physiological measures do not decrease in a linear pattern during recovery after exercise, and the same may be true for perceived fatigue. Second, while the other two studies employed a Pearson’s correlation analysis for each participant and then calculated the mean of all, our study utilized a within-subject correlation approach, which may be more appropriate for data with multiple repeated measurements. Nevertheless, the time course of perceived fatigue following exercise should be further investigated.

A new addition to the last phase of the study was the repetition of the first 5 min of the stepcycling test after participants had undergone a 10-minute recovery period. For the same time points and power output, there was a significant increase in perceived fatigue from the first to the second administration of the test, indicating that the scale is sensitive to accumulated fatigue from prior activities. Similar increases were observed for RPE and HR. However, it cannot be conclusively determined that the ROF scale would effectively capture such changes in fatigue throughout the remainder of the day following strenuous exercise.

At last, while the findings of this study demonstrate the successful translation and validation of the ROF scale into Portuguese, given the widespread use of Portuguese across multiple countries with distinct cultural and linguistic contexts, additional considerations are warranted. For instance, Brazil, the largest Portuguese-speaking country, exhibits linguistic variations that may influence the interpretation of certain scale descriptors. Similarly, countries such as Angola and Mozambique may require further cultural and linguistic adaptations to ensure the scale’s applicability. While the translation process aimed to maintain semantic and conceptual equivalence, some regional idiomatic expressions or terms may differ. Some nuances in the language highlight the need for further research to validate the scale in Portuguese from Brazil and in other regions where linguistic influences from local dialects or languages may affect its use. By expanding the reach of the ROF scale to include other Portuguese-speaking countries, this research has the potential to enhance the understanding and monitoring of fatigue in diverse contexts. As such, while the scale may be used in Portuguese-speaking countries in its current form, future studies should prioritize testing the scale’s reliability and validity in countries such as Brazil, Angola, and Mozambique, adapting it as necessary to address regional linguistic and cultural specificities.

### Limitations

While the main objectives of the study were achieved, there are some limitations which must be acknowledged. First, the participants employed in phases 2 and 3 were familiar with sport and exercise. While the ROF scale is intended to be used in varied scenarios and its validity was demonstrated in the original study, future research utilizing the Portuguese version of the scale should explore its use in different conditions and populations. Secondly, while in the last phase of the study it was demonstrated that the ROF scale is sensitive to accumulated fatigue, this should be further explored. Measurements after more prolonged periods of time or even throughout days and different time-points of the day would help strengthen these results. At last, this Portuguese version of the ROF scale should serve as a starting point for other Portuguese speaking countries. Further testing of its reliability and validity should be performed and changes made, as necessary, to address regional linguistic and cultural specificities of countries such as Brazil and Angola.

## Conclusions

The present study translated and validated the Rating of Fatigue (ROF) scale to Portuguese. By employing recommended cross-cultural adaptation methods, the scale and its accompanying instructions achieved high levels of comparability and interpretability relative to the original English version of the ROF scale. Face validity testing, consistent with methods used in previous validation studies, confirmed that the translated Portuguese version effectively measures fatigue.

The study demonstrated convergent validity between the ROF scale and physiological and perceptual measurements during a step cycling protocol and a subsequent 10-minute passive recovery period. Divergent validity was also maintained, as evidenced by the differentiation between ROF and RPE. This indicates that the Portuguese version of ROF retains the measurement properties observed in the original English version.

Notably, increases in perceived fatigue during the incremental exercise following an initial protocol and passive recovery suggest that the scale may detect accumulated fatigue from prior activities. However, further research is needed to explore this potential across a broader time frame. Results from face validity testing emphasize the importance of providing the ROF scale alongside the accompanying instructions sheet to aid in understanding and interpretation. Careful visual inspection and reading of the instructions are therefore recommended.

## Electronic Supplementary Material

Below is the link to the electronic supplementary material.

Supplementary Material 1: **Figure S1**. Portuguese version of the ROF scale.

## Data Availability

The datasets used and/or analyzed during the current study are available from the corresponding author on reasonable request.

## Abbreviations

HR: heart rate
ROF: rating of fatigue
RPE: rating of perceived exertion
SD: standard deviation
VO_2_: oxygen consumption
