# Supplementary material for: Translation and Validation of the Portuguese Version of the Rating-of-Fatigue Scale
Source: Sports Med Open. 2025 Feb 25;11:20. doi: 10.1186/s40798-025-00822-z (PMC11861487; doi:10.1186/s40798-025-00822-z)

A ESCALA DE CLASSIFICAÇÃO-DE-FADIGA E INSTRUÇÕES

A escala de classificação-de-fadiga (CDF) permitir-lhe-á avaliar o quão fatigado se sente. A escala poderá ser-lhe apresentada por uma outra pessoa ou, em algumas circunstâncias, poderá ser-lhe pedido que se autoavalie usando a mesma escala. Qualquer que seja o método utilizado, é importante que leia primeiro as seguintes orientações:

1. Por favor, familiarize-se com a escala, olhando, agora, atentamente para a mesma. Irá notar que a escala de CDF consiste em 11 pontos numéricos que vão de 0 a 10. Existem também cinco descritores e cinco diagramas que se destinam a ajudá-lo a compreender a escala e a fazer com que se classifique.
2. Quando lhe for apresentada a escala de CDF, por favor, inspecione cuidadosamente a escala antes de dar uma resposta numérica de 0 a 10. Tente sempre responder da forma mais honesta possível dando a classificação que melhor reflita a fadiga que sente naquele momento.
3. Tente não hesitar demasiado e certifique-se de que dá apenas UM número como resposta. Por exemplo, evite responder dando dois números tais como 'três ou quatro'.
4. Agora, por favor leia os seguintes exemplos do que algumas das classificações da escala de CDF significam:
   - Uma resposta de 0 indicaria que não se sente de todo fatigado. Um exemplo disto pode ser logo após acordar de manhã, depois de ter uma boa noite de sono. Agora, tente pensar numa ocasião semelhante no seu passado em que tenha experienciado as mais baixas sensações de fadiga e use isso como referência.
   - Uma resposta de 10 indicaria que se sente totalmente fatigado e exausto. Um exemplo disto pode ser não conseguir ficar acordado, talvez até tarde, à noite, mas poderia igualmente incluir situações como ‘sprintar’ até não poder fisicamente continuar mais. Mais uma vez, tente pensar num exemplo semelhante que tenha realmente experienciado no passado.

FADIGA TOTAL E
10 EXAUSTÃO – NÃO
RESTA NADA

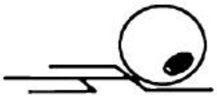

9

8

MUITO FATIGADO

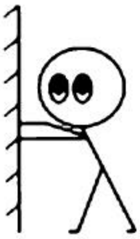

7

6

MODERADAMENTE
5 FATIGADO

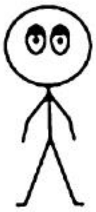

4

3

UM POUCO FATIGADO

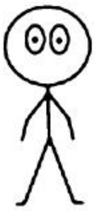

2

1

0 SEM SENSAÇÃO DE FADIGA

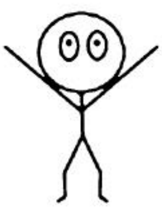

Supplement: Supplementary file 1 — Supplementary Material 1: Figure S1. Portuguese version of the ROF scale. [file 40798_2025_822_MOESM1_ESM.pdf]
